# Machine learning for RNA–small molecule interactions: methodologies, evaluation, and future directions

**DOI:** 10.1093/bib/bbag592

**Published:** 2026-11-05

**Authors:** Song Chen, Zhijian Huang, Yucheng Wang, Yahan Li, Guimei Liu, Yaw Sing Tan, Min Wu, Lei Deng

**Affiliations:** School of Computer Science and Engineering, Central South University, No. 932 Lushan South Road, Yuelu District, Changsha, Hunan 410083, China; School of Computer Science and Engineering, Central South University, No. 932 Lushan South Road, Yuelu District, Changsha, Hunan 410083, China; Institute for Advanced Intelligence and Computing, Agency for Science, Technology and Research (A^*^STAR), 1 Fusionopolis Way, #16‑16, Singapore 138632, Singapore; School of Computer Science and Engineering, Central South University, No. 932 Lushan South Road, Yuelu District, Changsha, Hunan 410083, China; Institute for Advanced Intelligence and Computing, Agency for Science, Technology and Research (A^*^STAR), 1 Fusionopolis Way, #16‑16, Singapore 138632, Singapore; Bioinformatics Institute, Agency for Science, Technology and Research (A^*^STAR), 30 Biopolis Street, #07‑01, Singapore 138671, Singapore; Institute for Advanced Intelligence and Computing, Agency for Science, Technology and Research (A^*^STAR), 1 Fusionopolis Way, #16‑16, Singapore 138632, Singapore; School of Computer Science and Engineering, Central South University, No. 932 Lushan South Road, Yuelu District, Changsha, Hunan 410083, China

**Keywords:** RNA–small molecule interactions, binding site prediction, binding affinity prediction, binding pose prediction, deep learning, RNA drug discovery

## Abstract

RNA is emerging as a compelling therapeutic target due to its rich structural diversity and central involvement in gene regulation, splicing, translation, and cellular signaling. Alongside advances in structural biology, computational approaches, especially machine learning and deep learning, have transformed the systematic analysis of RNA–small molecule interactions. In this paper, we provide a comprehensive survey of computational methods for RNA–small molecule interaction prediction, with a particular emphasis on three core tasks: binding site prediction, binding preference prediction, and binding pose prediction. We first summarize the data resources commonly used to support these tasks and outline molecular representations that capture RNA structural features and the chemical properties of small molecules. We then review a spectrum of computational approaches for the above three tasks, ranging from statistical methods and traditional machine learning to deep learning. We further evaluate representative methods for each task, providing insights into their respective strengths and limitations. Finally, we highlight open challenges in current research on RNA–small molecule interactions, including limited data availability, the black-box nature of the models, and the growing need for generative modeling. We discuss emerging directions such as data augmentation, model interpretability, and RNA-targeted small-molecule design as promising pathways to address these challenges and advance rational RNA-targeted drug discovery.

## Introduction

RNA has emerged as a promising class of therapeutic targets owing to its structural diversity and central roles in gene expression, splicing, translation, and signal regulation [1]. While traditional drug discovery has largely focused on proteins, RNA molecules exhibit unique structural plasticity that enables them to fold into complex secondary and tertiary architectures [2]. These conformations form well-defined pockets and grooves that can bind small molecules, offering opportunities for precise modulation of RNA functions [3]. Exploiting these RNA–small molecule interactions has facilitated the regulation of diverse biological processes, including riboswitch-mediated control, alternative splicing, and protein–RNA interactions. Riboswitches, as structured regulatory elements within messenger RNAs (mRNAs), provide compelling examples of RNA–small molecule interactions by directly binding small-molecule ligands and regulating mRNA expression through ligand-induced alternative RNA structures [4, 5]. Furthermore, small molecules that target RNA have emerged as promising therapeutic agents. Notable examples of RNA-targeting small molecules include ribocil, which targets the FMN riboswitch to disrupt bacterial metabolism [6], risdiplam, which promotes exon inclusion in SMN2 pre-mRNA to treat spinal muscular atrophy [7], and zotatifin, which inhibits eIF4A helicase to suppress oncogenic translation [8]. These cases underscore the growing therapeutic relevance of RNA–small molecule interactions.

Despite growing interest in RNA as a therapeutic target, systematic exploration of RNA–small molecule interactions remains hindered by limitations in data availability and quality. Experimental approaches including X-ray crystallography, nuclear magnetic resonance spectroscopy, and cryo-electron microscopy have provided high-resolution structural insights into individual RNA–small molecule complexes. However, these methods are costly and low-throughput, making experimentally resolved RNA–small molecule structures exceedingly rare compared with the extensive repertoire of protein–ligand complexes [9]. Consequently, existing databases such as the Protein Data Bank (PDB) [10], HARIBOSS [11], RNAglib [12], R-SIM [13], and R-BIND [14], capture only a tiny fraction of the RNA structural and chemical space. To gain a deeper understanding of how small molecules recognize and modulate RNA structures for drug discovery [15], computational and machine learning approaches have emerged as essential tools to bridge this gap.

Computational approaches therefore play an essential role in addressing the limitations of experimental characterization and enabling systematic analysis of RNA–small molecule interactions. Leveraging available sequence, structural, and chemical information, these methods facilitate the identification of small-molecule binding regions. They further enable the prediction of binding preferences across diverse chemical scaffolds and the estimation of ligand orientations and conformations within RNA pockets. Traditional docking and physics-based simulations have contributed valuable mechanistic insights into RNA–small molecule recognition. For example, molecular dynamics and umbrella-sampling simulations of adenine riboswitches have shown how ligand binding stabilizes terminal helices and long-range loop-loop contacts, offering mechanistic intuition about RNA conformational switching beyond static structures [16]. However, their broad applicability remains constrained by high computational cost, limited scalability, and the highly flexible nature of RNA molecules [17]. To address these challenges, machine learning–based approaches have emerged as compelling alternatives that learn patterns directly from RNA and small molecule interaction data. Rather than relying on explicit energy functions or exhaustive conformational sampling, these methods exploit sequence-, structure-, and chemistry-aware features to capture statistical patterns underlying RNA–small molecule interactions. Deep learning models further extend this paradigm by integrating multi-scale molecular information across RNA and small molecule domains, enabling end-to-end modeling of complex and nonlinear interaction mechanisms. As a result, these data-driven approaches offer improved predictive accuracy and scalability [18], while supporting systematic exploration of the chemical and structural features for RNA at a genomic scale.

Recently, several reviews [19–22] have examined computational methods for RNA–small molecule interaction prediction from different perspectives. Among them, Gao *et al*. [19] focus on RNA–small molecule binding site prediction, highlighting the methodological transition from physics-based approaches to artificial intelligence (AI)-driven models and summarizing benchmark comparisons. Meanwhile, the other three reviews [20–22] survey computational methods across a broader range of tasks, including binding site prediction, binding preference prediction, and binding pose prediction. Notably, Zhuo *et al*. [21] further report benchmark results generated in [23] and [24], covering binding site prediction on the TE18 dataset and binding preference prediction on the UNK96 dataset.

In this survey, we present a comprehensive and systematic re-examination of machine learning methods for predicting RNA–small molecule interactions. Compared with existing reviews, our work offers three key contributions. First, we introduce a unified framework that integrates data curation, feature representation, computational modeling, and systematic evaluation, as illustrated in Fig. 1. This framework provides a clearer and more actionable roadmap for advancing machine learning and AI in RNA-targeted therapeutics. Second, beyond summarizing existing methods, we examine annotation quality and benchmark redundancy in commonly used binding-site datasets (e.g. Train60, Test18 and Test9) [25, 26]. For example, after accounting for overlap with Train60 and Test18, only one independent RNA (1Y26) remained in Test9. Test9 was therefore not used for evaluation. Test17 was derived from Test18 by removing the incorrectly annotated entry 6EZ0. Third, we conduct a more comprehensive evaluation of various models across different prediction tasks, data settings, and evaluation protocols. This broader assessment well complements existing task-specific surveys and provides practical guidance for practitioners in processing the data and selecting appropriate methods for real-world applications.

**Figure 1:**
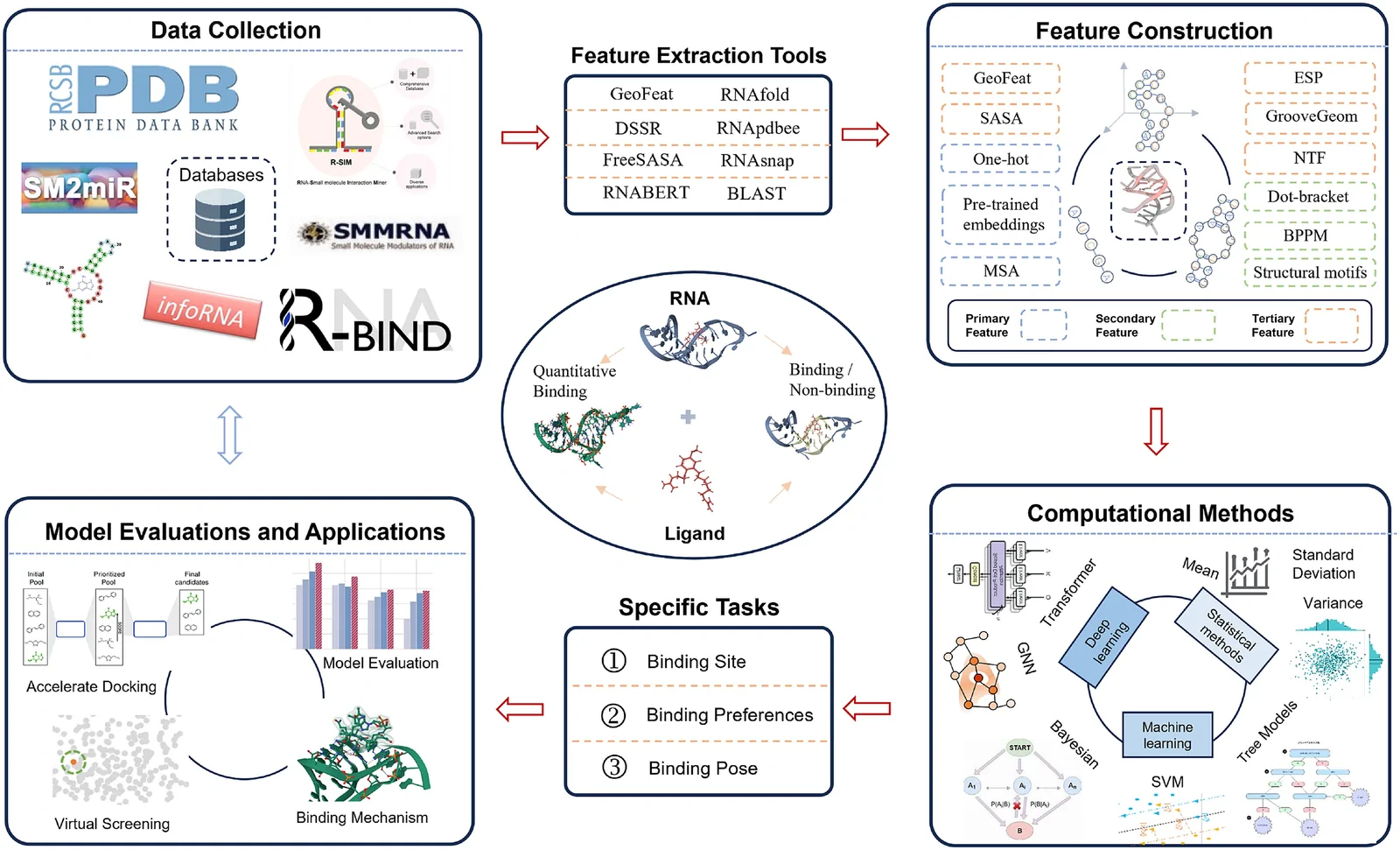
Overview of the computational workflow for RNA–small molecule interaction modeling. The pipeline covers data collection, feature construction for RNA and small molecule representations, computational modeling with statistical, traditional machine learning, and deep learning methods, and final model evaluation and application in RNA-targeted small molecule discovery.

## Datasets

Reliable and well-annotated datasets form the cornerstone of machine learning and deep learning approaches for modeling RNA–small molecule interactions. These datasets provide the empirical basis for training, validating, and benchmarking computational models. Depending on the level of interaction information they contain, current datasets can be broadly grouped according to their relevance to binding site prediction, binding preference prediction, and binding pose estimation, as summarized in Table 1. These tasks help in understanding RNA–ligand interactions: binding site prediction identifies where ligands bind on RNA, binding preference prediction ranks ligands by affinity or binding score, and binding pose estimation predicts the 3D orientation of the ligand within the binding site.

**Table 1:** Representative benchmarks and datasets for RNA–small molecule modeling. ①, ②, and ③ denote binding site prediction, binding preference prediction, and binding pose prediction tasks, respectively

Datasets	Task	Size	Description	Website/source
PDB [10]	① ② ③	251 558	Total PDB entries; RNA–small molecule complexes require task-specific filtering	https://www.rcsb.org/
HARIBOSS [11]	① ③	1000	Curated RNA binding site annotations	https://hariboss.pasteur.cloud/
rnaglib [12]	① ③	8441	RNA 3D motifs and site descriptors	https://github.com/cgoliver/rnaglib
R-SIM [13]	②	2501	Validated RNA–small molecule affinity	https://web.iitm.ac.in/bioinfo2/R_SIM/
PDBbind [27]	① ③	234	RNA–small molecule complexes with affinities	http://www.pdbbind-plus.org.cn/
SMMRNA [28]	②	770	Small molecule–miRNA regulatory interactions	http://www.smmrna.org/
RNALigands [29]	②	841	Motif-based RNA–small molecule interaction set	https://rnajournal.cshlp.org/content/28/2/115
R-BIND [14]	②	153	Bioactive RNA-targeting small molecules	https://pubs.acs.org/doi/10.1021/acschembio.9b00578
SM2miR [30]	②	4989	Small molecule–miRNA effect profiles	http://www.jianglab.cn/SM2miR/
Inforna [31, 32]	②	1936	Motif–ligand mapping from secondary structure	https://disney.scripps.ufl.edu/software/
NALDB [33]	②	3610	Affinity-annotated nucleic acid ligand entries	https://academic.oup.com/database/article/doi/10.1093/database/baw002/2630176

Datasets for binding sites primarily characterize the specific RNA regions or residues involved in ligand binding. The PDB [10] remains the most comprehensive source of experimentally determined structures of biomacromolecules, containing 251 558 entries as of 31 March 2026. However, because the PDB is not specifically curated for RNA–small molecule interactions, several specialized databases have been developed to extract and annotate relevant entries. For example, HARIBOSS [11] and RNAglib [12] curate and annotate RNA 3D structures, providing residue-level annotations and structural descriptors that facilitate the extraction of features for machine learning.

Datasets describing RNA–small molecule binding preferences can be broadly divided into affinity-oriented and preference-oriented resources, depending on whether they emphasize quantitative binding strength or binding selectivity across RNA motifs and small molecule classes. Affinity-oriented datasets provide experimentally measured binding affinities (e.g. K$_{i}$, K$_{d}$, IC$_{50}$). PDBbind [27] provides binding affinity values and structural information for RNA–small molecule complexes from PDB. R-BIND [14] and R-SIM [13] further provide experimentally validated RNA-binding small molecules with associated affinity measurements, integrating sequence, structure, and physicochemical information for modeling structure–affinity relationships. The Nucleic Acid Ligand Database (NALDB) [33] contains 3610 nucleic acid–ligand entries. It provides physicochemical descriptors, affinity information, and conformers for virtual screening. By contrast, preference-oriented datasets capture which classes of small molecules tend to interact with specific RNA motifs or regulatory elements. R-BIND [14] catalogs bioactive ligands that selectively target non-rRNA. In R-BIND 2.0 [34], RNA structural motifs are analyzed to identify new targeting strategies. SM2miR [30] records experimentally validated interactions between 151 small molecules and 747 microRNAs (miRNAs) across 17 species, summarizing their up- or down-regulatory effects. Inforna 2.0 [32] mines RNA secondary structure motifs within target RNAs and compares them with known motif–ligand interactions, now encompassing 1936 entries involving 244 unique compounds and 1331 motifs, with enhanced search and similarity tools. Finally, RNALigands [29] integrates data from PDB, R-BIND, and Inforna to compile 841 motif-based RNA–ligand interactions, serving as a comprehensive reference for exploring RNA structural motifs and their binding preferences.

Datasets for binding pose prediction emphasize the 3D conformations of RNA–ligand complexes, capturing the spatial orientation of ligands within RNA binding pockets. The majority of pose-level data for RNA–ligand complexes are derived from the PDB [10]. These high-resolution entries provide the structural ground truth necessary for docking validation and pose prediction tasks.

## Features for RNAs and small molecules

Effective feature representation is the cornerstone of modeling for RNA–small molecule interactions, as it defines how biological and chemical entities are encoded numerically for downstream predictive tasks. In this section, we summarize the strategies for transforming RNA and small molecules into machine-interpretable features. For RNA, various approaches extract features from primary, secondary, and tertiary structures to capture hierarchical folding patterns and physicochemical context. For small molecules, both traditional physicochemical descriptors and modern graph- or Transformer-based embeddings are used to represent molecular topology, geometry, and electronic properties. Together, these representations define the input space for machine learning and deep learning models in tasks such as RNA–small molecule interaction prediction.

### RNA features

As summarized in Table 2, RNA features can be defined at multiple levels of structural abstraction, including primary, secondary, and tertiary structures.

**Table 2:** Summary of RNA and small-molecule feature representations and corresponding extraction tools

**Input**	**Feature level**	**Feature type**	**Representative tools**
RNA	Primary	One-hot encoding	—
		MSA	MUSCLE [35], T-Coffee [36], Clustal Omega [37], MAFFT [38]
		Pretrained embeddings	RNA-FM [39], ERNIE-RNA [40], RNABERT [41], RiNALMo [42]
	Secondary	Dot-bracket notation	ViennaRNA [43], mfold [44], RNAstructure [45], RNApdbee [46], MXfold2 [47]
		BPPM	ViennaRNA [43], RNAstructure [45]
		Non-Watson–Crick base-pairing	DSSR [48], RNApdbee [46]
		Structural motifs	RNApdbee [46], MXfold2 [47]
	Tertiary	SASA (solvent accessibility)	FreeSASA [49], NACCESS [50], RNAsnap2 [51]
		Network topology attributes	MetaNet [52]
		Electrostatic potential	APBS [53], MSMS [54]
		Groove width	Materials Studio [55], DSSR [48]
		Torsion angles	Materials Studio [55], DSSR [48]
Small molecule	Topological	Fingerprints (ECFP, MACCS)	RDKit [56], Open Babel [57], BIOVIA Discovery Studio [58]
		Molecular graph representation	RDKit [56], DeepChem [59], DGL-LifeSci [60]
	Physicochemical	Basic molecular properties	PaDEL-Descriptor [61], Mordred [62], RDKit [56]
		Atom-level features	RDKit [56], DeepChem [59]
		Intermolecular interaction descriptors	RDKit [56], Open Babel [57]
	Learned	SMILES/SELFIES embeddings	ChemBERTa [63], MegaMolBART [64], MolFormer [65], MolMVC [66]

At the primary structure level, features are derived directly from RNA sequences. A common representation is one-hot encoding, which encodes each nucleotide as a binary vector and provides a simple yet interpretable input for downstream models. Beyond this, multiple sequence alignments (MSA) capture evolutionary conservation and co-variation among homologous sequences, offering richer contextual information than single-sequence representations. MSA features can be generated by tools like MUSCLE [35], T-Coffee [36], Clustal Omega [37], or MAFFT [38]. More recently, sequence-based RNA pretrained models, such as RNA-FM [39], ERNIE-RNA [40], RNABERT [41], and RiNALMo [42], leverage transformer architectures to learn contextual embeddings from large-scale RNA sequence corpora.

At the secondary structure level, features capture RNA folding patterns and base-pairing interactions. Common representations include the dot-bracket notation, which encodes paired and unpaired nucleotides in a compact symbolic form. In this scheme, dots represent unpaired residues, while matching brackets denote base-paired positions, allowing the entire secondary structure topology to be expressed as a linear string. For instance, given an RNA segment forming a hairpin, the dot–bracket string (((...))) indicates that the first three nucleotides are paired with the last three, while the three nucleotides in between remain unpaired, corresponding to a loop. Owing to its simplicity and compatibility with downstream computational pipelines, the dot-bracket format has become a standard intermediate representation in RNA informatics. Another frequently used representation is the base pairing probability matrix (BPPM), which quantifies the likelihood of base-pair formation across the sequence and provides a probabilistic view of structural variability. For an RNA of length *L*, the BPPM is an *L × L* matrix in which each cell stores the probability that two nucleotide positions form a base pair. As an example, Fig. 2 shows a BPPM generated by RNAfold from the chain A sequence of PDB entry 1AJU [43]. In this representation, stronger signals indicate nucleotide pairs with higher pairing probabilities. Non-Watson–Crick base-pairing and other noncanonical contacts are often described using the Leontis–Westhof geometric classification, which provides a structural vocabulary for RNA base-edge interactions important in tertiary folds and ligand-recognition motifs [67]. In addition, structural motifs such as hairpins, internal loops, bulges, and junctions serve as higher-order descriptors that summarize recurrent secondary structure elements, as shown in Fig. 3. These features can be derived using computational tools such as ViennaRNA [43], mfold [44], RNAstructure [45], RNApdbee [46], or MXfold2 [47], which predict secondary structures based on thermodynamic modeling or deep learning. Collectively, these representations provide a detailed view of the local and global pairing topology, facilitating the identification of potential ligand-binding regions in RNA molecules.

**Figure 2:**
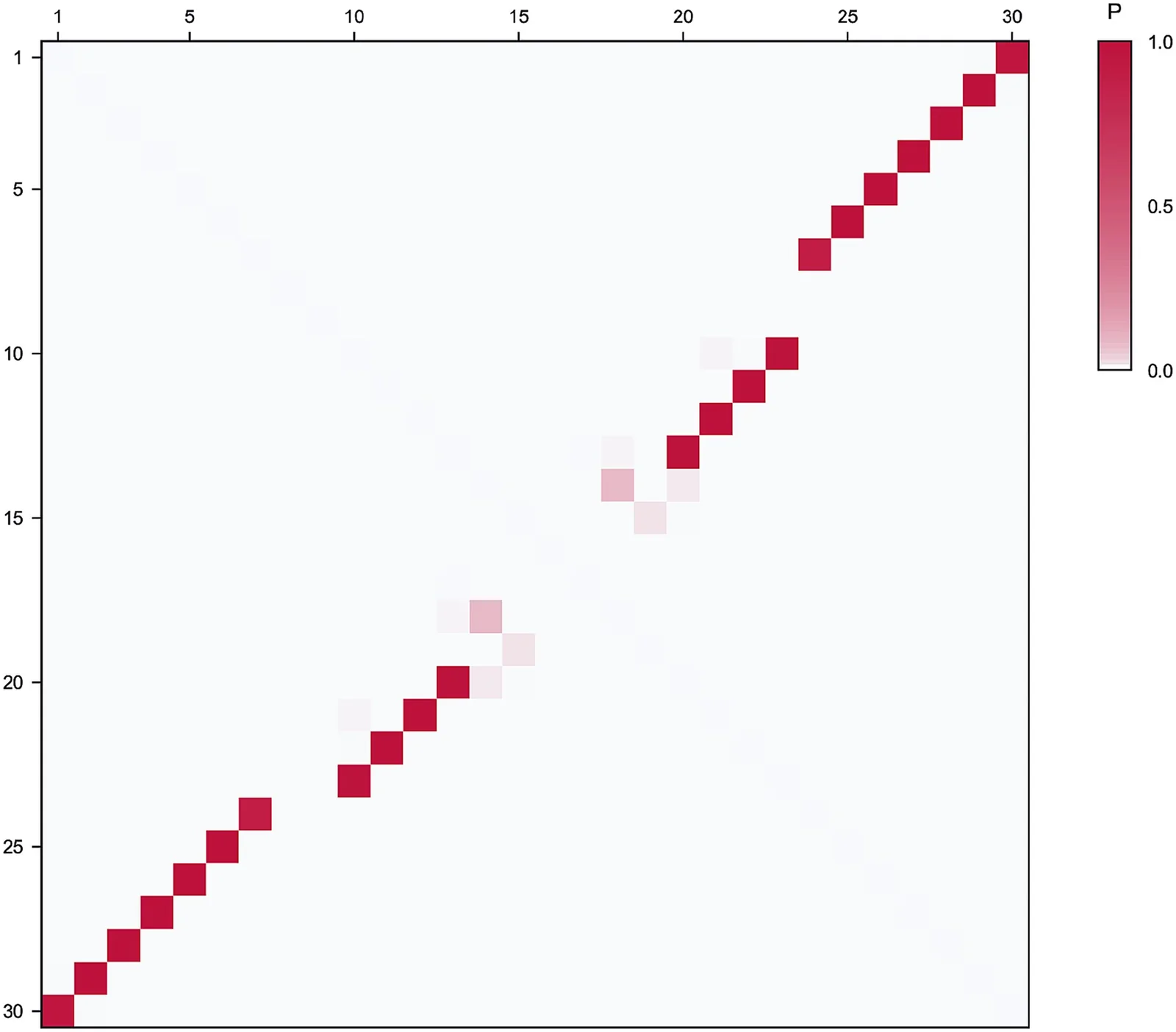
Example BPPM generated from the chain A sequence of PDB entry 1AJU using RNAfold. Each cell represents the base-pairing probability between two nucleotide positions, with stronger signals indicating more probable base-pairing contacts.

**Figure 3:**
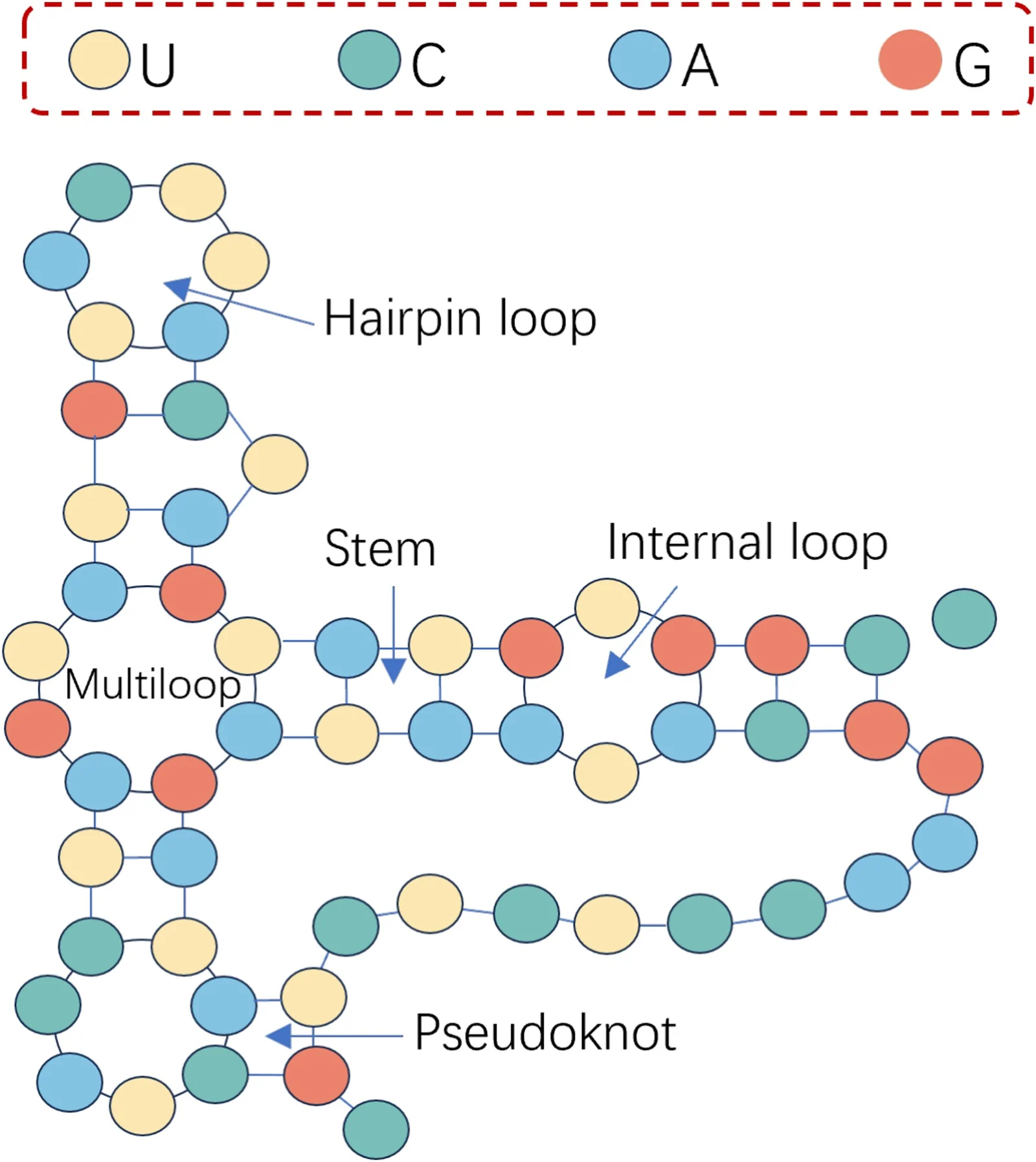
Representative RNA secondary structure motifs. These motifs include hairpin loops, internal loops, bulges, and junction-like elements. Paired stems and unpaired nucleotides together define local architectures that can form ligand-recognition environments.

At the tertiary structure level, RNA features describe the geometric and physicochemical properties critical for small molecule binding. The solvent-accessible surface area (SASA), which quantifies nucleotide exposure to solvent and indicates potential binding accessibility, can be calculated using tools such as FreeSASA [49], NACCESS [50], or RNAsnap2 [51]. The electrostatic potential, reflecting charge distribution patterns that determine electrostatic complementarity with small molecules, is commonly obtained through APBS (Adaptive Poisson–Boltzmann Solver) [53] while the molecular surface can be computed by MSMS [54]. The groove widths, capturing variations in major and minor groove geometry that influence small molecule binding, can be measured using DSSR [48]. Similarly, torsion angles, which define backbone and base conformations shaping RNA flexibility and 3D architecture, can also be extracted by DSSR [48].

### Features for small molecules

For small molecules, the choice of feature representation critically influences a model’s capacity to generalize across chemical space, as shown in Table 2. Topological descriptors such as extended-connectivity fingerprints and MACCS keys encode the presence of specific molecular substructures and connectivity patterns. These fingerprints can be efficiently computed using cheminformatics toolkits including RDKit [56], Open Babel [57], or commercial suites such as BIOVIA Discovery Studio [58].

Beyond topology, physicochemical descriptors provide quantitative measures of fundamental molecular properties. Commonly used features include molecular weight, logP, pKa, topological polar surface area, and the counts of hydrogen-bond donors and acceptors, which together characterize drug-likeness and chemical reactivity. Atom-level properties such as atom type, valence, hybridization state, aromaticity, and partial charge further enrich structural detail, while intermolecular interaction descriptors-including hydrogen-bond interactions, pi–pi stacking, van der Waals forces, and electrostatic interactions-capture the binding tendencies that often govern RNA–small molecule recognition. These categories of features can be computed using widely used tools such as PaDEL-Descriptor [61], Mordred [62], RDKit [56], and DeepChem [59].

With the rise of deep learning in cheminformatics, learned molecular representations have become increasingly prominent. Graph-based approaches treat atoms as nodes and bonds as edges, enabling graph neural networks, message-passing neural networks, and attention-based architectures to learn hierarchical molecular features from structures generated through toolkits such as RDKit [56], DeepChem [59], or DGL-LifeSci [60]. In parallel, SMILES- and SELFIES-based language models such as ChemBERTa [63], MegaMolBART [64], MolFormer [65], and MolMVC [66] represent molecules as chemical sequences with contextual embeddings that reflect underlying chemical grammar, reactivity patterns, and functional group context. Further, DrugLLM [68] leverages powerful language models for drug design by learning molecule modifications from chemical texts, exhibiting strong few-shot learning capabilities. Mol-L2 [69] integrates both chemical text knowledge and molecular structure through a two-stage training pipeline, generating text-enhanced molecular embeddings for various downstream tasks. By integrating topological, physicochemical, and learned descriptors within these computational frameworks, modern small molecule representations offer a robust and versatile foundation for downstream applications such as affinity prediction, virtual screening, and structure-oriented generative molecular design.

## Computational methods for RNA–small molecule interaction prediction

With the extracted representations of RNA and small molecules, computational methods can be employed to predict RNA–small molecule interactions. In this review, we categorize these approaches into three major task families: RNA–small molecule binding site prediction, binding preference prediction, and binding pose prediction. As illustrated in Fig. 4, these three tasks together provide a comprehensive view of RNA–small molecule interactions, and each task is intrinsically connected to the others. Binding pose defines the spatial orientation and atomic contacts of a ligand, thereby guiding the identification of plausible binding sites and informing binding preference by modulating interaction strength and specificity. Conversely, the strength of binding preference affects whether a ligand is likely to engage with an RNA target at all, thereby impacting the feasibility of both binding site and binding pose prediction. Moreover, accurate binding site information offers structural constraints that guide pose generation by narrowing the search space to physically meaningful regions. Through these interdependencies, binding site prediction, preference estimation, and pose modeling collectively form an integrated framework for understanding RNA–small molecule interactions.

**Figure 4:**
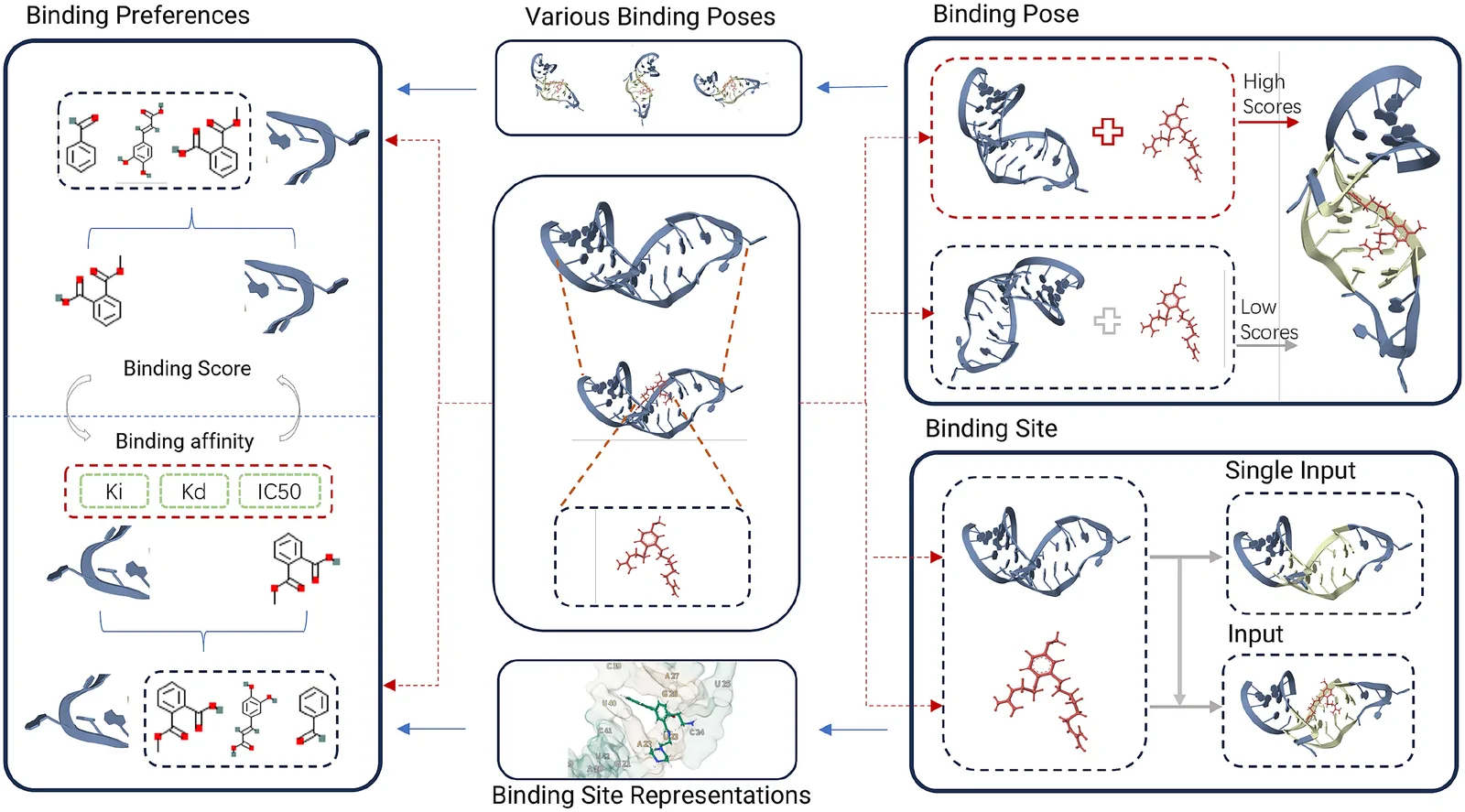
Illustration of three core computational tasks in RNA–small molecule interaction modeling: (a) *Binding site prediction*, which identifies the nucleotide residues that constitute potential ligand-binding regions in an RNA structure; (b) *Binding pose prediction*, which determines the 3D orientation and conformation of a small molecule when bound to the RNA pocket; and (c) *Binding preference prediction*, which evaluates the relative binding affinity or likelihood of interaction between an RNA target and diverse candidate ligands.

### Binding site prediction

Predicting RNA binding sites is a central problem in understanding RNA–small-molecule interactions and enabling rational RNA-targeted drug design. Binding site prediction operates at the nucleotide level and aims to identify the nucleotide residues that form the ligand-binding region. Computational approaches for this task have progressed from early statistical analyses to traditional machine learning models and, more recently, to deep learning methods that emphasize representation learning from sequence and structural data.

Early statistical approaches identify RNA binding sites by analyzing geometric and interaction patterns derived from experimentally resolved RNA structures. These methods typically assume that binding nucleotides exhibit distinctive spatial or topological properties. Rsite [70] and Rsite2 [71] detect functionally important nucleotides by analyzing distance distributions, with Rsite focusing on tertiary structures and Rsite2 extending the approach to secondary structures. RBind [23] models RNA tertiary structures as graphs and identifies binding residues based on deviations in network connectivity. MetalionRNA [72] uses distance- and angle-dependent statistical potentials derived from RNA–metal ion complexes to identify preferred ion-binding geometries. While these approaches are interpretable and require limited training data, they rely heavily on high-quality experimental structures and fixed assumptions about binding patterns, which restrict their applicability and predictive accuracy.

Traditional machine learning methods extend statistical models by incorporating more diverse feature sets and supervised learning frameworks. RNAsite [25] combines sequence-derived and tertiary structure features and applies a random forest classifier to predict binding residues. RNet [73] further introduces topological descriptors from RNA tertiary structures and uses ensemble learning to improve prediction stability. NABS [74] provides an integrative platform for predicting binding sites across RNA, DNA, and protein targets by combining structural, sequence, and interaction preference features across multiple PDB-derived datasets, together with post-processing modules to refine predictions. Despite improved performance over statistical methods, these models depend on handcrafted features, which limits scalability and makes them sensitive to feature selection.

Deep learning approaches have become the dominant paradigm by enabling automatic feature learning from raw sequence and structural inputs. Early models such as RLBind [26] employ convolutional neural networks to integrate local and global structural information. MultiModRLBP [75] extends this idea through a multimodal architecture that combines 3D nucleotide features, relational topology via graph convolution, and sequence semantics from the RNA language model RNABert [41]. This design improves discrimination between RNAs with identical sequences but different tertiary structures. However, these models still require explicit structural inputs and are constrained by the availability and reliability of RNA 3D structures. Subsequent studies focus on capturing spatial and geometric properties of RNA binding environments. ZeSTa [76] transforms local RNA surface regions into structured feature maps that integrate sequence, secondary structure, and geometric information, which are classified using a convolutional neural network. $\mathrm{BiteNet}_{N}$ [77] applies 3D convolutional networks to voxelized RNA structures to predict binding probability maps and pocket centers. RLBSIF [78] adopts a surface-based learning strategy, representing RNA pockets as meshes annotated with geometric and electrostatic features and using a MaSIF-based network for binding site prediction. These geometry-aware models improve spatial resolution and interpretability but incur high computational cost and remain sensitive to structural noise. To better integrate spatial and sequential context, hybrid architectures have been proposed. The RLsite model [79] combines energy-based 3D representations with sequence- and structure-derived features to capture both local interactions and long-range dependencies.

Recent graph and geometric deep learning methods further incorporate contextual embeddings derived from RNA foundation models to enhance binding site prediction. CoBRA [80] demonstrates that residue-level embeddings from pretrained RNA language models can achieve competitive binding site prediction performance without explicit structural information. GATRsite [81] represents RNA molecules as graphs that combine structural descriptors with pretrained embeddings and applies attention-based graph neural networks to improve robustness to noisy or predicted structures. The method proposed by Sun *et al*. [82] further integrates ERNIE-RNA embeddings with tertiary structure features using graph attention and feature fusion modules. RNABind [83] adopts equivariant graph neural networks to jointly model sequence context and 3D geometry, achieving strong performance across benchmarks. Building on these advances, MVRBind [84] introduces a multi-view graph convolutional framework that jointly models sequence, secondary structure, and tertiary structure representations. By enabling multi-scale feature interaction through message passing and attention, MVRBind improves robustness across diverse RNA conformations, including apo structures, and addresses limitations related to structural dependency and generalization in previous methods.

### Binding preference prediction

Binding preference prediction operates at the RNA–ligand pair level and aims to estimate or rank the strength of interactions between RNA molecules and small-molecule ligands. Computational approaches to binding preference prediction are generally divided into classification-based and regression-based methods. Classification-based approaches simplify the task by formulating it as a binary classification, distinguishing binding from nonbinding pairs. For example, ZHMol-RLinter [24] employs a hierarchical random forest to aggregate nucleotide-level features for binary prediction, while DistRMI [85] leverages deep neural representations of RNA loop motifs and small molecules together with spatial distance information to improve prediction accuracy. Although these models are robust and computationally efficient, they do not capture graded binding strength. In contrast, regression-based methods model the interaction strength as a continuous variable and are broadly categorized into affinity-based and score-based approaches. Affinity-based methods aim to predict experimentally measured binding affinities, whereas score-based methods aim to predict relative binding affinities.

Affinity-based regression models aim to predict quantitative measures such as dissociation constants or binding free energies. Early approaches relied on statistical models and traditional machine learning using handcrafted descriptors. RSAPred [86] applies RNA-subtype-specific linear regression to sequence-derived descriptors, yielding interpretable predictions of experimental binding affinity. SPrank [87] combines knowledge-based statistical potentials with a random forest regressor to estimate binding free energy from RNA–small molecule complexes. These early methods emphasize interpretability and physical intuition but are constrained by limited feature expressiveness and simplified interaction modeling. Recent affinity prediction methods increasingly rely on deep learning to learn joint RNA–small molecule representations. DeepRSMA [88] uses cross-attention to fuse RNA and small molecule graph representations for affinity regression. RLaffinity [89] predicts binding affinity directly from 3D complexes using contrastive pretraining and 3D convolutional networks. RLASIF [90] encodes RNA–small molecule interface surfaces into geometric fingerprints for deep regression. EMMPTNet [91] integrates multiple RNA sequence views with small molecule sequence and physicochemical features to improve stability and interpretability. DLRNA-BERTa [92] combines pretrained RNA and molecular language models with cross-attention to predict affinity from sequence and SMILES. BioLLMNet [93] similarly fuses large language model (LLM) embeddings for RNA and ligands using a gating mechanism for end-to-end affinity prediction. While these deep models achieve higher accuracy and generalization, they often require large training datasets and may suffer from limited interpretability.

Score-based regression models predict continuous binding scores that reflect relative binding preference rather than experimental affinity values. These approaches are particularly suited for virtual screening and large-scale small molecule prioritization tasks. Among score-based methods, deep learning approaches dominate due to their ability to learn structural and chemical representations automatically. RNAmigos [94] encodes RNA binding sites as base-pairing graphs and ranks ligands based on learned structural embeddings. RNAmigos2 [95] extends this framework with dual encoders trained on large-scale docking and experimental data, enabling fast and accurate small molecule enrichment. RNAsmol [96] predicts binding scores directly from RNA sequence and small molecule graphs using attention-based fusion. SMRTnet [97] integrates RNA sequence and secondary structure features with molecular language models for structure-free score prediction. DeepRNA-DTI [98] is a sequence-based deep learning model that leverages pretrained RNA and molecule embeddings with multitask learning to jointly predict binding score and binding site. GerNA-Bind [99] jointly models RNA sequence, secondary structure, and 3D geometry with molecular representations to predict binding specificity and uncertainty. Overall, score-based models are efficient and scalable but primarily provide relative rather than physically interpretable interaction measures.

### Binding pose prediction

Binding pose prediction focuses on determining the 3D orientation and conformation of small molecules when bound to RNA. Accordingly, methods in this category are interpreted at the conformational level, including pose generation, docking, and pose-quality evaluation. Compared with binding site or affinity prediction, pose prediction requires modeling both geometric complementarity and physicochemical interactions between RNA and ligands at atomic resolution.

LigandRNA [100] is an early knowledge-based scoring method for RNA–ligand interactions that uses an anisotropic statistical potential derived from 251 experimentally resolved complexes to rank ligand poses based on distance- and angle-dependent interaction preferences. Subsequently, machine learning–based scoring functions have been increasingly explored for pose evaluation and rescoring. RNAPosers [101] encodes each ligand pose using a composite fingerprint that describes the local RNA environment around ligand atoms and applies random forest classifiers to distinguish native-like poses from decoys. Similarly, AnnapuRNA [102] employs k-nearest-neighbor models and deep neural networks trained on coarse-grained, distance- and angle-based RNA–ligand contact descriptors to estimate the likelihood that a pose corresponds to a native binding mode. More recently, RmsdXNA [103] formulates pose evaluation as a regression task and predicts the root-mean-square deviation (RMSD) between predicted and experimental poses using distance-based interaction fingerprints combined with XGBoost regression [104], enabling quantitative pose quality assessment across RNA, DNA, and hybrid nucleic acid–ligand complexes.

In contrast to scoring and rescoring approaches, the following methods focus on nucleic acid–ligand docking. AutoDock [105] is one of the earliest and most widely used docking programs, originally developed for protein targets. It employs a Lamarckian genetic algorithm coupled with a semi-empirical free energy function to sample ligand conformations within predefined binding sites and has been extensively applied to RNA–ligand docking. Dock6 [106], also initially designed for protein–ligand docking, was later adapted to RNA–ligand systems through modified sampling strategies and scoring protocols, and is frequently used as a general-purpose docking engine in nucleic acid–ligand studies. Subsequently, rDock [107] introduced a fast genetic algorithm–based docking framework with scoring functions tailored for nucleic acids and support for pharmacophoric and positional restraints, enabling efficient RNA-focused virtual screening. More recently, RLDOCK [17] proposed RNA-oriented docking protocols that emphasize RNA-specific interaction patterns during pose generation and evaluation, improving docking performance on RNA–ligand complexes. NLDock [108] further advanced nucleic acid–ligand docking by integrating the knowledge-based scoring function ITScoreNL into a shape-matching docking framework, allowing efficient sampling of rigid and flexible ligand poses and accurate ranking of RNA–ligand binding modes. PLANTS [109] adopts an optimization-driven strategy, combining ant colony optimization with continuous local search to efficiently sample and refine ligand binding poses guided by empirical scoring functions. In the nucleic acid–ligand docking benchmark of Jiang *et al*. [110], LeDock was evaluated together with PLANTS and rDock, and these three programs showed the strongest performance for binding pose prediction.

In addition to docking algorithms for RNA, several structure-based ligand pocket detection methods originally developed for proteins have been adapted for RNA applications. For example, fPocket [111], a well-established protein pocket detection tool, has been successfully extended to identify binding cavities in RNA structures [112]. More recently, fPocketR was introduced as an RNA-oriented variant that enables systematic detection and characterization of ligand-binding pockets in RNA [113, 114].

## Model evaluation

To systematically assess current computational methods for RNA–small molecule interactions, we organize the evaluation around three task families: RNA binding site prediction, binding preference prediction, and binding pose prediction. These three tasks are evaluated at different levels, with binding site prediction at the residue or nucleotide level, binding preference prediction at the RNA–ligand pair level, and binding pose prediction at the conformational or atomic level. The evaluation was based on several key metrics: Accuracy, which measures the overall correctness of the model; Precision, which calculates the proportion of true positive predictions among all positive predictions; Recall, reflecting how many actual positive instances the model successfully predicted; F1 Score, which balances precision and recall through their harmonic mean; Area Under the Curve (ACC), indicating the model’s ability to distinguish between classes; Matthews Correlation Coefficient (MCC), providing a balanced measure of prediction quality, accounting for both true and false positives and negatives; Balanced Accuracy (BACC), which adjusts accuracy for class imbalance; and Pearson Correlation Coefficient (PCC), measuring the linear correlation between predicted and actual values. Among these metrics, success rate requires a task-specific interpretation. In binding-preference ranking, success means that the true ligand is among the top 10 candidates. In binding-preference classification, success means that a true binding pair is classified as binding. In binding-pose prediction, success means that the top-ranked ligand pose has an RMSD *≤ 2.0* Å from the experimental pose.

### RNA binding site prediction

Binding site prediction aims to identify RNA residues that interact with small molecules. To achieve this, residue-level features are extracted from 1D/2D/3D RNA data. Sequence features provide residue-level information for identifying sequence patterns associated with ligand binding, while secondary-structure features capture local RNA structural organization and residue accessibility. Three-dimensional structural features provide spatial context for identifying binding pockets and characterizing ligand–residue contacts, offering more direct structural information for residue-level binding site prediction.

#### Refining datasets for binding site prediction

Test18 [25] and Test9 [26] have been widely used to evaluate RNA–small molecule binding site prediction methods, yet both exhibit critical issues that undermine their reliability for rigorous model evaluation.

Test18 was introduced by RNAsite and has since been widely adopted as a common benchmark in subsequent studies, including RNet [73], RLBind [26], MultiModRLBP [75], RNABind [83], and MVRBind [84]. In Test18, one entry (PDB ID: 6EZ0) does not contain a genuine small-molecule ligand. Instead, it includes an unnatural nucleotide (U37) that is incorporated into the RNA chain during *in vitro* transcription. This does not represent an exogenous ligand-binding event and therefore introduces misleading supervision signals for learning-based models, as illustrated in Fig. 5.

**Figure 5:**
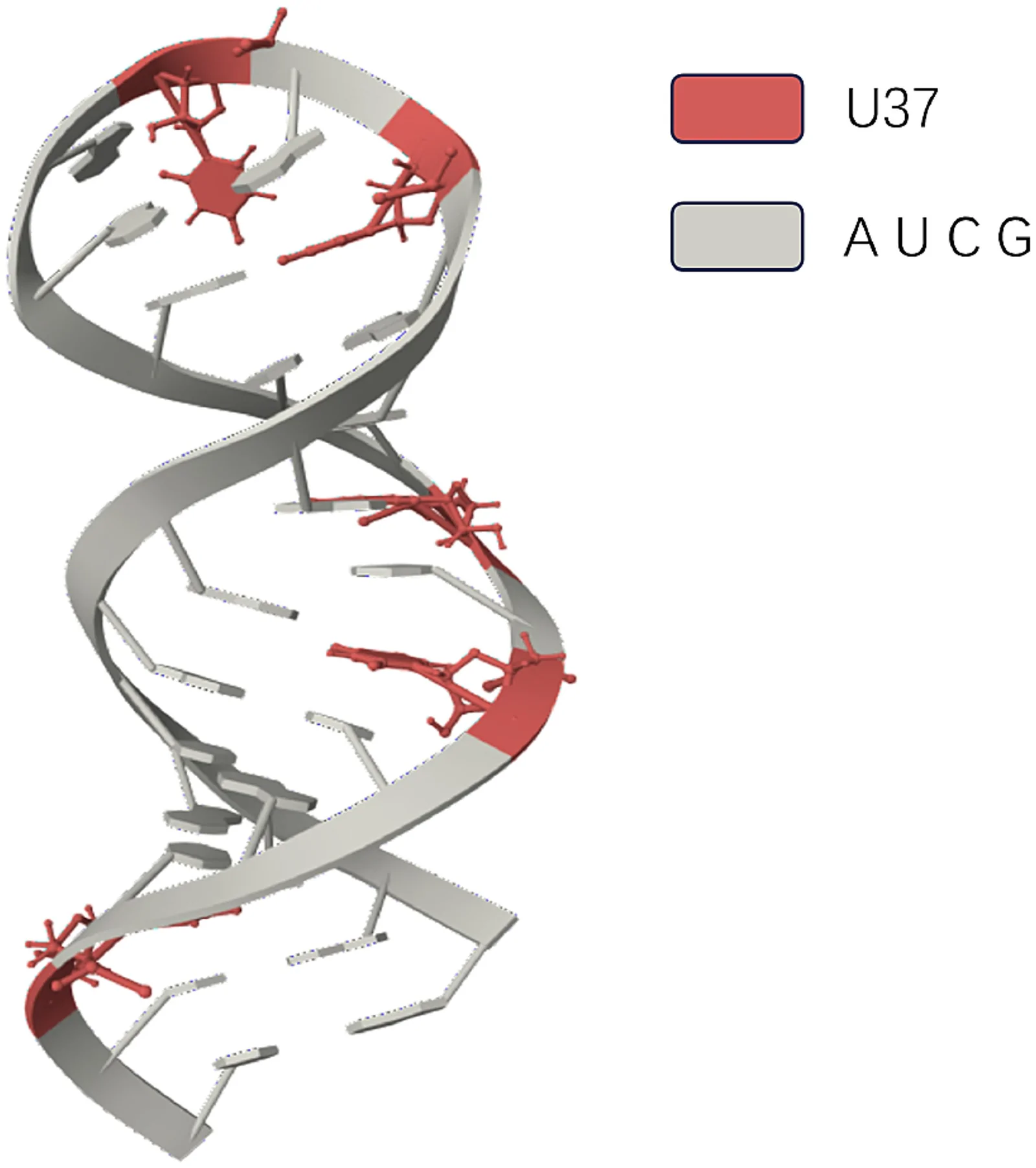
The problematic entry in Test18: 6EZ0 contains an unnatural nucleotide (U37) rather than a small-molecule ligand.

Test9 [26] exhibits substantial sequence and structural redundancy with Train60. If highly similar RNA entries occur in both sets, such data leakage may lead to overestimated model generalization. To further substantiate the extent of redundancy, Tables 3 and 4 provide representative examples of structural and sequence similarities between Test9 and Train60, respectively. As shown in Table 3, multiple RNA pairs exhibit exceptionally high TM-scores, far exceeding the commonly adopted redundancy threshold of 0.3. Notably, the RNA pair (3GX2, 3GX3) achieves a TM-score of 0.987, indicating nearly identical 3D structures. Similarly, other pairs such as (2GDI, 4NYB) and (3D2X, 4NYB) also demonstrate very high structural similarity, with TM-scores above 0.79. Even the lowest reported TM-score in the table (0.308 for 1UUI versus 1UUD) still meets the redundancy criterion. These results clearly indicate that a substantial portion of Test9 shares highly similar structural folds with training samples, undermining its ability to evaluate structural generalization.

**Table 3:** Structural similarity between Test9 and Train60 measured by RNA-align (TM-score)

**Test9 RNA**	**Train60 RNA**	**TM-score**
1PBR	1BYJ	0.452
1UUI	1UUD	0.308
2GDI	4NYB	0.794
3D2X	4NYB	0.794
3GX2	3GX3	0.987

**Table 4:** Representative sequence similarity between Test9 and Train60 based on CD-HIT-EST clustering. The suffix following the underscore indicates the RNA chain ID

**Test9 RNA**	**Train60 RNA**	**Identity (%)**
3GX2_A	3GX3_A	100%
1UUI_B	1UUD_B	100%
1PBR_A	1BYJ_A	100%

Consistent with the structural analysis, Table 4 shows that several Test9 RNA sequences are identical to those in Train60, with sequence identity reaching 100%. For instance, the pairs (3GX2_A, 3GX3_A), (1UUI_B, 1UUD_B), and (1PBR_A, 1BYJ_A) are clustered together by CD-HIT-EST under a 30% identity threshold, indicating that they are not merely similar but effectively duplicates at the sequence level. This level of redundancy suggests that models evaluated on Test9 may encounter nearly identical sequences during training, leading to overly optimistic performance estimates. After applying both independence criteria, four Test9 RNA chains remain. Three of them (i.e. 1NEM, 1Q8N, and 2TOB) also occur in Test18 [25]. Consequently, only 1Y26 is independent of both Train60 and Test18. Test9 is unsuitable for the subsequent benchmark evaluation, and it is not used in the quantitative comparison.

Test17 is constructed exclusively from Test18 [25]. PDB entry 6EZ0 is excluded because it contains an unnatural nucleotide rather than a separate small-molecule ligand. Test17 contains no samples from Test9. It is used for the subsequent binding-site benchmark comparison. As shown in Table 5, we summarize the dataset statistics before and after curation.

**Table 5:** Statistics of the training dataset (Train60), the original test set (Test18), and the annotation-corrected test set (Test17)

**Dataset**	**RNA chains**	**Nucleotides**	**Pos.**	**Neg.**
Train60	60	3054	950	2104
Test18 (original)	18	610	207	403
Test17 (refined)	17	583	191	392

#### Results on refined datasets

Using the refined Test17 benchmark, we systematically re-evaluated various models as shown in Table 6. In particular, Rsite, Rsite2, and RBind are deterministic methods under fixed input structures and therefore produce a single performance value for each metric. For the rest deep learning-based methods, results are reported as mean *± * standard deviation across repeated evaluations.

**Table 6:** Model comparison of representative models for RNA binding site prediction on the Test17 benchmark

Model	Accuracy	Precision	Recall	F1 Score	AUC	MCC	BACC
Rsite [70]	0.647	0.381	0.126	0.189	0.513	0.040	0.513
Rsite2 [71]	0.597	0.345	0.257	0.294	0.510	0.021	0.510
RBind [23]	0.690	0.596	0.162	0.255	0.554	0.179	0.554
RNAsite [25]	*0.680 ± 0.005*	*0.550 ± 0.029*	*0.128 ± 0.025*	*0.207 ± 0.033*	*0.686 ± 0.013*	*0.136 ± 0.022*	*0.538 ± 0.009*
RNet [73]	*0.709 ± 0.002*	*0.602 ± 0.007*	*0.330 ± 0.007*	*0.426 ± 0.006*	*0.714 ± 0.001*	*0.274 ± 0.007*	*0.612 ± 0.003*
RLBind [26]	*0.657 ± 0.043*	*0.493 ± 0.056*	*0.533 ± 0.091*	*0.507 ± 0.022*	*0.689 ± 0.016*	*0.250 ± 0.040*	*0.626 ± 0.016*
RNABind [83]	*0.686 ± 0.029*	*0.536 ± 0.040*	*0.571 ± 0.057*	*0.552 ± 0.036*	*0.729 ± 0.019*	*0.312 ± 0.054*	*0.658 ± 0.027*
MultiModRLBP [75]	*0.699 ± 0.040*	*0.575 ± 0.072*	*0.492 ± 0.089*	*0.525 ± 0.044*	*0.722 ± 0.039*	*0.314 ± 0.060*	*0.649 ± 0.027*
MVRBind [84]	*0.698 ± 0.014*	*0.561 ± 0.022*	*0.516 ± 0.091*	*0.535 ± 0.052*	*0.713 ± 0.033*	*0.315 ± 0.046*	*0.654 ± 0.029*

Statistical methods showed limited predictive power, with Rsite [70] achieving an accuracy of 0.647 and an F1 score of only 0.189. Its reliance on Euclidean distance extrema proved inadequate for flexible RNA backbones, where functional pockets are not always defined by purely geometric features. Rsite2 [71], which integrates both secondary and tertiary structure distances, exhibited slightly higher recall of 0.257 but comparable overall performance, suggesting that geometric extrema alone remain insufficient for capturing functionally relevant sites. RBind [23], which identifies binding residues based on closeness and degree values above a mean-plus-standard-deviation threshold, improved precision to 0.596 but suffered from low recall of 0.162, as the fixed cutoff was unable to adapt to structural diversity among RNAs.

Machine learning methods further improved predictive performance on Test17. RNAsite [25], which combines degree, closeness, Euclidean distance, and MSA features within a random forest framework, reached an accuracy of 0.680 and an AUC of 0.686. This indicates that integrating topological and evolutionary information can enhance site-level discrimination, although the reliance on manually designed features limits adaptability to complex spatial relationships. RNet [73], which applies ensemble learning to a richer set of topological and physicochemical descriptors, achieved an accuracy of 0.709, an F1 score of 0.426, and an AUC of 0.714. These results suggest that RNet’s feature selection effectively captures biologically relevant interaction patterns. However, the handcrafted nature of its representations constrains its ability to model nonlinear structural dependencies inherent in RNA–small molecule recognition. Notably, the evaluation metrics of machine learning-based methods remain relatively stable. Their reliance on handcrafted features and fixed model designs results in predictable performance but limits the modeling of complex nonlinear interactions.

Deep learning models generally achieved better and more balanced performance than traditional machine learning approaches on Test17, owing to their ability to integrate diverse feature types through end-to-end learning. RLBind [26], which combines biochemical, structural, and evolutionary features using convolutional neural networks applied to local and global RNA contexts, achieved precision of 0.493 and recall of 0.533, with an F1 score of 0.507 and AUC of 0.689. RNABind [83] performed similarly, reaching an accuracy of 0.686 and AUC of 0.729, demonstrating that combining sequence and structure encoders can effectively capture both spatial and contextual dependencies. MultiModRLBP [75] extended this idea by integrating relational graph convolutional networks, a pretrained RNABert [41] module for capturing contextual semantics, and ELBSF and MFFusion components for fine-grained structural feature fusion. It achieved an AUC of 0.722, indicating the benefit of multi-scale and multi-modal representation learning. MVRBind [84] integrates multi-view graph representations of RNA primary, secondary, and tertiary structures. On Test17, it attains an accuracy of 0.698 and an AUC of 0.713. Overall, deep learning–based models consistently outperform traditional machine learning approaches on Test17 by leveraging end-to-end integration of features. However, their performance varies more across different runs, reflecting increased sensitivity to training strategies, data partitioning, and the quality of learned structural representations.

### RNA binding preference prediction

RNA–small molecule binding preference prediction is evaluated from two complementary perspectives. First, regression-based models are assessed for their ability to predict interaction strength, including affinity-based models and binding score–based models. Affinity-based models are evaluated using sequence-based and structure-based methods to examine the impact of different molecular representations on quantitative prediction. Sequence-based methods extract features from RNA sequences and secondary structures, while structure-based methods further incorporate 3D features capturing the spatial geometry and physicochemical complementarity of RNA–ligand contacts, including hydrogen bonding, stacking, electrostatic, and van der Waals interactions. Second, classification based models are evaluated for their ability to discriminate binding from nonbinding RNA–ligand pairs, reflecting their effectiveness in qualitative interaction assessment.

Affinity-based prediction represents a major direction in RNA–small molecule interaction modeling. Existing studies typically construct datasets from PDBbind [27] or related affinity resources and develop models for predicting binding preference. These methods can be broadly categorized into sequence-based and structure-based approaches as mentioned above, and representative methods are evaluated in Fig. 6.

**Figure 6:**
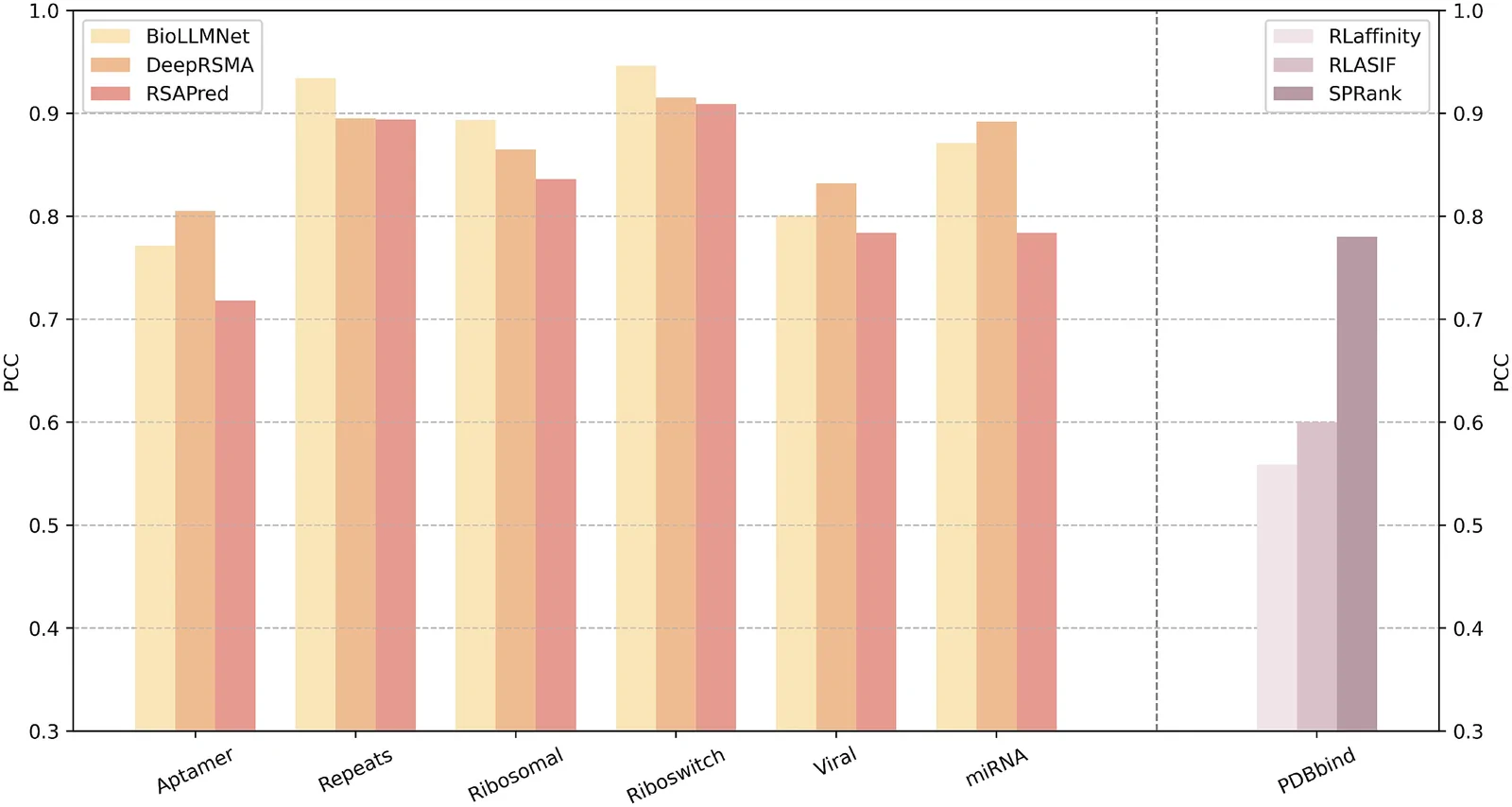
Performance comparison of RNA–ligand affinity prediction models. The left panel shows sequence-based models on the R-SIM dataset across six RNA subtypes (aptamer, miRNA, repeats, ribosomal RNA, riboswitch, and viral RNA), whereas the right panel shows structure-based models on the PDBbind NL2020-derived affinity dataset.

Sequence-based affinity prediction methods infer RNA–small molecule binding tendencies from primary sequence composition, motif patterns and secondary-structure signals, and are typically benchmarked on the R-SIM dataset, which spans diverse RNA subtypes such as aptamers, repeats, ribosomal RNAs, riboswitches, viral RNAs, and miRNAs. As illustrated in Fig. 6, RSAPred [86] provides a foundational baseline by combining handcrafted sequence descriptors with subtype-aware linear modeling. It performs reliably on repeats and riboswitches, achieving correlation values of 0.894 and 0.909, but its accuracy is lower for aptamers at 0.718 and viral RNAs at 0.784, reflecting the challenges posed by highly variable or structurally diverse RNA families. DeepRSMA [88] builds upon these ideas by integrating sequence embeddings with predicted secondary structure information and graph-based RNA topology, coupled with ligand features through a cross-attention mechanism. This multi-view design yields consistently stronger performance across most R-SIM subtypes. DeepRSMA [88] reaches 0.895 for repeats, 0.865 for ribosomal RNAs, and 0.915 for riboswitches, outperforming RSAPred [86] in each of these categories. It also achieves the highest values among the three models for viral RNAs at 0.832 and for miRNAs at 0.892, indicating that incorporating structural proxies and graph-based signals enhances its capacity to model affinity determinants. BioLLMNet [93] further extends sequence-based prediction by introducing LLM embeddings trained on broad RNA corpora. These contextualized representations provide a flexible and scalable way to extract sequence-derived features without manual descriptor engineering. BioLLMNet [93] achieves the strongest overall performance across most R-SIM subtypes, reaching 0.934 for repeats, 0.893 for ribosomal RNAs, and 0.946 for riboswitches. It also performs competitively on aptamers with a value of 0.771 and on viral RNAs with 0.800, although these categories show slightly higher values for DeepRSMA [88]. Overall, the results demonstrate that LLM-based representations capture subtle sequence and motif signals that are highly informative for affinity prediction. Together, these findings highlight a clear progression from descriptor-based baselines to multi-view neural architectures and LLM embeddings. Performance improvements across the models reflect increasingly expressive representations of sequence-derived interaction cues and their ability to generalize across heterogeneous RNA subtypes.

In contrast to sequence-based approaches, structure-based affinity prediction leverages the 3D geometry of RNA–ligand complexes to capture spatial complementarity and physicochemical interaction patterns. These methods are typically evaluated on the affinity dataset, which contains 95 RNA–ligand complexes with experimentally measured binding affinities from PDBbind NL2020 data set [90]. As summarized in Fig. 6, three representative models—RLaffinity [89], SPRank [87], and RLASIF [90]—illustrate distinct modeling paradigms and performance characteristics. RLaffinity [89] utilizes a voxelized 3D pocket representation combined with convolutional architectures. For a fair RNA-specific evaluation, we re-assessed RLaffinity [89] on the affinity dataset after removing DNA-containing entries included in its original training set, yielding a Pearson correlation coefficient of 0.56. SPRank [87] adopts a knowledge-based statistical potential that incorporates distance-dependent atomic preferences, combined with a random forest regressor to refine affinity prediction. It achieves the strongest performance among the three methods, reaching a PCC of 0.78. RLASIF takes a geometric perspective by encoding molecular surface curvature, electrostatics and local patch features using geodesic convolution, achieving a PCC of 0.60 on the same dataset. Collectively, these structure-based methods highlight the importance of explicit 3D information for estimating RNA–ligand affinity. Voxelized representations, statistical potentials, and geometric surface descriptors capture complementary aspects of RNA binding energetics, offering multiple avenues for modeling the structural determinants of RNA–small molecule recognition.

Within regression-based binding preference prediction, we next focus on the evaluation of binding score–based models, which assign continuous scores to RNA–ligand pairs to reflect relative binding preferences rather than true binding affinities. Such models are particularly advantageous for large-scale virtual screening or early-stage drug discovery scenarios where experimentally measured affinities are sparse or unavailable. To support such evaluation, the UNK96 [24] benchmark was derived from surveying RNA–small molecule complexes deposited in the PDB between August 2020 and June 2024, during which 180 RNA chains binding small molecules were identified and subsequently curated into a set of 96 representative RNA–ligand pairs. Because many binding score–based methods do not perform explicit binary classification, their performance is evaluated using a ranking-based criterion. For the three ranking methods, success rate is the percentage of test cases in which the true binding small molecule appears among the top 10 candidates. Candidates are ranked by the Euclidean distance between predicted and candidate MACCS fingerprints. A smaller distance indicates greater similarity [24]. As illustrated in Table 7, their performance varies substantially on the UNK96 benchmark [24]. RNAmigos [94] represents RNA binding sites as augmented base-pairing graphs and employs a relational graph convolutional network to predict the molecular fingerprints of potential ligands. RNAmigos achieves the lowest success rate among the three ranking methods, as its generalization is limited by the predefined binding sites and the relatively small number of experimentally characterized RNA–ligand complexes. R-BIND adopts a cheminformatics-driven strategy that matches ligands to recurrent RNA structural motifs curated from experimental literature, achieving a success rate of 26.0%. RNALigands [29] further utilizes RNA secondary-structure motifs combined with database-driven similarity search, producing a success rate of 22.9% on the same benchmark.

**Table 7:** Comparison of success rates on the UNK96 benchmark. For ranking methods, success means that the true ligand is retrieved within the top 10. For ZHMol-RLinter, success means that a true binding pair is classified as binding

**Method**	**Success rate (%)**
Regression methods: success if ranked in top-10.	
RNAmigos [94]	15.6
R-BIND [14]	26.0
RNALigands [29]	22.9
Classification methods: success if classified as binding.	
ZHMol-RLinter [24]	77.1

In addition, we assess classification-based models by examining their performance in discriminating binding versus nonbinding RNA–ligand pairs. For ZHMol-RLinter [24], the success rate is 77.1%. This value is the percentage of true binding pairs classified as binding. It describes performance under the classification protocol.

### RNA binding pose prediction

RNA binding pose prediction seeks to determine the 3D configuration of a ligand within an RNA binding pocket. In this task, sequence and secondary-structure features provide complementary information about RNA identity and local structural constraints, while 3D complex structures provide the spatial reference required to assess ligand placement within RNA binding pockets. Experimentally resolved RNA–ligand complexes therefore serve as critical references for evaluating whether predicted poses reproduce native-like ligand conformations and interactions. Current computational approaches generally fall into two categories. One class relies on scoring functions that assess the quality of candidate poses based on geometric complementarity or interaction energetics. The other class uses docking frameworks that explicitly sample ligand conformations and placements to generate plausible binding poses. In the following sections, we summarize representative methods from both categories and examine their performance on commonly used RNA–ligand benchmarking datasets. Specifically, four test sets are widely used for pose evaluation: the Yan set, the Ruiz set, the Chen set, and the Philips set. Following the comparison reported by RmsdXNA [103], each complex is evaluated using its highest-ranked prediction. A prediction is successful when the ligand RMSD is no greater than 2.0 Å from the experimental pose. The success rate is the percentage of benchmark complexes that meet this criterion.

Scoring-function-based approaches constitute some of the earliest methods for RNA–ligand binding pose prediction and remain widely used due to their computational efficiency and interpretability. On the Philips benchmark (Table 8), LigandRNA [100] and DrugScoreRNA [115] achieve success rates of 35.7% and 31.0%, respectively. Their relatively modest performance can be attributed to the reliance on fixed statistical potentials or protein-derived scoring terms, which are insufficient to capture the high conformational flexibility, noncanonical base interactions, and heterogeneous electrostatic environment characteristic of RNA binding pockets. RmsdXNA [103] improves the success rate to 37.8% by incorporating local geometric alignment features, enabling better recognition of near-native poses when local structural similarity is preserved. However, because it primarily focuses on local geometry, its ability to model long-range or globally coordinated RNA–ligand interactions remains limited. SPRank [87] achieves the highest success rate of 45.2% among scoring-function methods. By combining knowledge-based atomic statistical potentials with a random forest model, it integrates both local interaction patterns and broader pocket-level descriptors. This hybrid design allows SPRank to partially compensate for the limitations of purely physics- or statistics-based scoring functions, resulting in more robust discrimination between native-like and decoy poses across diverse RNA–ligand complexes.

**Table 8:** Top-1 pose success rates of scoring-function methods on the Philips set. A prediction is successful when the highest-ranked pose has ligand RMSD *≤ 2.0* Å from the experimental pose

**Method**	**Success rate (%)**
LigandRNA [100]	35.7
DrugScoreRNA [115]	31.0
RmsdXNA [103]	37.8
SPRank [87]	45.2

Docking-framework methods constitute the second major category of RNA binding pose prediction. As shown in Table 9, NLDock [108] achieves success rates of 73%, 64%, 68%, and 71% on the Yan, Ruiz, Chen, and Philips datasets, respectively, indicating relatively consistent performance across diverse benchmarks. This suggests that incorporating nucleic acid oriented statistical potentials within the docking workflow can improve pose ranking across different RNA ligand systems. rDock [107] and DOCK6 [106] display intermediate performance, with success rates ranging from 57% to 70% for rDock and from 38% to 58% for DOCK6 across the four datasets. Their results are comparatively stable between benchmarks, which may be attributed to more constrained sampling strategies and electrostatic treatments that reduce the generation of highly unfavorable poses. However, the absence of fully RNA specific interaction modeling may limit their ability to further improve discrimination between near native and decoy conformations. AutoDock [105] shows lower success rates overall, with values between 16% and 38% across the same benchmarks. While its sampling procedure is capable of generating diverse ligand conformations, the scoring function originally developed for protein ligand docking may not adequately capture RNA specific interaction patterns, such as noncanonical base contacts and the distinct electrostatic environment of RNA pockets. This discrepancy likely affects pose ranking accuracy rather than pose generation itself.

**Table 9:** Top-1 pose success rates (%) of docking-framework methods on four standard RNA–ligand pose-prediction benchmarks. A prediction is successful when the highest-ranked pose has ligand RMSD *≤ 2.0* Å from the experimental pose

**Method**	**Yan**	**Ruiz**	**Chen**	**Philips**
NLDock [108]	73	64	68	71
AutoDock [105]	34	16	38	33
rDock [107]	66	57	70	57
DOCK 6 [106]	58	38	57	48

## Discussions and future directions

Computational models for RNA–small molecule interactions typically address three main tasks: binding-site prediction, affinity or preference prediction, and binding pose prediction. These tasks correspond to the fundamental questions in RNA-targeted drug discovery: where a ligand can bind on the RNA structure, which ligands are most likely to bind, and how the molecular interaction occurs at the structural level. Rather than functioning independently, these tasks are inherently interconnected and form a coherent workflow in computational drug discovery. Binding-site prediction narrows the structural search space by identifying potential pockets in RNA molecules. Affinity or preference prediction then evaluates large libraries of compounds to prioritize molecules likely to interact with these sites. Finally, pose prediction characterizes the detailed binding configuration between RNA and ligand, providing structural insight into the interaction. Together, these three components form an integrated pipeline to improve ligand prioritization and guide rational drug design [116].

Despite increasing progress, significant challenges remain in modeling RNA–small molecule interactions, including limited data availability [11], the black-box nature of the models [117], and the growing need for generative modeling [118]. To address these challenges, several research directions are emerging, including data augmentation, model interpretability, and RNA-targeted small-molecule design to advance RNA-targeted drug discovery.

### Data augmentation

Current research on RNA–small molecule interactions is limited by the scarcity of high-quality RNA structural data, as only a small set of experimentally resolved RNA–small molecule complexes are available in the PDB [10, 27]. This limitation is particularly severe for tasks that require broad coverage of conformational variability, binding pocket diversity, or small molecule–induced structural changes. To address this limitation, a common strategy is data augmentation.

One augmentation strategy is to expand structural coverage using high-confidence computationally predicted RNA structures. When strict quality control and confidence evaluation are applied, predicted structures can substantially enlarge the available structural space. Integrating these predicted structures with experimentally determined complexes allows models to learn from a wider range of conformations and binding environments, thereby improving robustness and generalization [119]. MVRBind shows that multi-view GCN and multi-scale fusion enable effective binding site prediction even from predicted RNA tertiary structures. More recently, an approach has been developed to predict RNA–small molecule interactions based on RNA sequence and secondary structure information, without the need for RNA tertiary structures [97]. In addition to dataset expansion, augmentation can be performed on existing structures through controlled conformational perturbations. For example, computationally generated structural variants that moderately deviate from native folds can be sampled, and perturbations can be introduced to simulate alternative base-pairing or base-stacking arrangements. These strategies expose models to diverse structural variations and help them better capture the inherent flexibility of RNA.

The above augmentation strategies are particularly beneficial for deep learning models, which often require large and diverse training data to learn robust representations [120]. Furthermore, data-efficient learning approaches [121], including few-shot learning, self-supervised learning, and transfer learning, can be leveraged to improve model performance when labeled data are limited. Transfer learning can be applied via a unified modeling framework by pretraining a protein–ligand model and fine-tuning it on RNA–small molecule datasets to adapt shared interaction patterns to RNA structures. [122] By combining structural augmentation with data-efficient learning strategies, models can better generalize from limited RNA–small molecule interaction data.

### Model interpretability

Deep learning models have demonstrated strong predictive accuracy for RNA–small molecule interaction prediction. However, they often function as black boxes and fail to reveal the physicochemical principles governing RNA–small molecule interactions, such as hydrogen bonding, *π *–*π * stacking, electrostatic interactions with the phosphate backbone, metal-ion coordination, and solvation effects. Therefore, it is highly desirable to develop deep learning models that not only achieve high predictive accuracy but also provide mechanistic interpretation of RNA–small molecule interactions.

Addressing this challenge requires modeling strategies that explicitly encode intermolecular interaction mechanisms. One promising direction is the use of interaction-aware architectures that incorporate physics-inspired components, such as differentiable layers modeling hydrogen bonding, electrostatics, stacking energetics, or metal-ion stabilization [123]. Attention mechanisms aligned with interpretable structural features—such as RNA base edges, grooves, or phosphate groups—can highlight binding hotspots and key atomic contacts that determine specificity. In addition, hybrid frameworks that combine machine-learned representations with physically motivated descriptors or energy decomposition terms can provide interpretable scoring functions linked to recognizable biochemical interactions. Visualization tools, including interaction attribution maps and force-decomposition saliency analyses, can further support expert interpretation by connecting predictions with concrete molecular interactions.

LLMs may also contribute by integrating structural predictions with biochemical knowledge, generating human-readable explanations of predicted RNA–small molecule interactions and assisting researchers in interpreting model outputs [124]. Overall, improving interpretability of RNA–small molecule interaction models requires the integration of physically meaningful interaction representations, interpretable architectures, and explanatory tools. Such advances will help transform predictive models from black-box predictors into mechanistically informative systems that support rational small molecule optimization and RNA-targeted drug discovery.

### RNA-targeted small molecule design

Protein-targeted small molecule design [118, 119, 125] has progressed rapidly in the past few years, while we lack generative models that can directly support small molecule design for RNA targets. With increasing studies on RNA–small molecule interactions, our understanding of their binding mechanisms, such as binding sites and binding poses, has significantly improved. At the same time, the amount of available data on RNA–small molecule interactions continues to grow. These advances make it increasingly feasible to design and generate novel small molecules that specifically target RNA.

Future research should focus on structure-based design approaches [126] that condition molecular generation on the structural context of RNA binding pockets and adapt the generated molecules to diverse RNA motifs and topologies. Such models should translate RNA structural features, such as local motif geometry, pocket topology, and multi-domain organization, into constraints that guide *in silico* molecule generation. They should also support design modes relevant to practical discovery workflows, including pocket-conditioned generation, RNA motif–guided scaffold elaboration, and compatibility-driven optimization for complex RNA architectures. In addition, RNA–small molecule complex structures are still limited. Hence, developing data-efficient AI methods for RNA-targeted small molecule design [126] is another important research direction.

## Conclusion

In this review, we surveyed recent machine learning approaches for modeling RNA–small molecule interactions, focusing on binding site prediction, binding preference prediction, and binding pose prediction. By organizing existing methods along the modeling pipeline from data construction and molecular representation to predictive modeling and evaluation, we highlight how data-driven approaches have advanced the systematic analysis of RNA–small molecule recognition. Our analysis indicates that recent improvements are largely driven by deep learning models that integrate sequence, structural, and chemical information, enabling more accurate and scalable prediction across diverse RNA targets and small-molecule chemotypes. At the same time, we emphasize that dataset quality and evaluation protocols play a critical role in determining reported performance. Annotation errors, redundancy, and inappropriate data splitting in commonly used benchmarks can substantially bias model assessment and obscure true generalization ability.

Despite notable progress, challenges remain, including limited high-quality data, insufficient interpretability of predictive models, and the lack of dedicated generative frameworks for RNA-targeted small molecule design. Addressing these issues through improved data curation, robust evaluation strategies, and biologically grounded modeling will be essential for translating computational advances into practical RNA-targeted drug discovery.

**Key Points**

- We present a unified and systematic framework that integrates data curation, feature representation, computational modeling, and evaluation, offering a clear roadmap for advancing machine learning in RNA–small molecule interactions.
- We identify common issues in widely used RNA–small molecule datasets, including annotation errors and redundancy, and analyze their potential impact on model training and evaluation.
- We perform a comprehensive evaluation of models across tasks, data settings, and protocols, providing practical guidance for real-world applications.
- Future progress will depend on improved data curation, greater model interpretability, and the development of RNA-oriented generative models to support rational drug discovery.

## Data Availability

The codes and datasets are available online at https://github.com/cschen-y/rna-small-molecule-review.
